# Driving under the influence of alcohol and drugs on e-scooters: a toxicological perspective of public health and road safety in Leipzig, Germany

**DOI:** 10.1007/s00414-026-03877-z

**Published:** 2026-06-17

**Authors:** Pascal Städter, Delen Kuntze, Jan Dreßler, Susen Becker

**Affiliations:** 1 Institute of Forensic Medicine, Medical Faculty, University of Leipzig, Leipzig, Germany; 2 Institute of Forensic Medicine, Universität Leipzig, Johannisallee 28, 04103 Leipzig, Germany

**Keywords:** E-scooter, Traffic control, Intoxicating substances, Driving under influence of alcohol, Driving under influence of drugs

## Abstract

**Background:**

Since October 2021, it has been possible in Leipzig to rent e-scooters and to participate actively in road traffic. Since then, a large number of traffic offenses has been recorded.

**Aim of the study:**

This study examines alcohol and drug use among e-scooter riders in Leipzig and the surrounding area — particularly in relation to the new legal regulations. The accidents are analyzed in detail.

**Materials/methods:**

A total of 411 traffic offenses recorded by the police between October 2021 and June 2024 were retrospectively analyzed.

**Results:**

The study cohort consisted of 91.2% men. Of all rides under the influence of intoxicating substances, 65.9% took place in Leipzig. Differentiation of the traffic offenses revealed that 67.2% were administrative traffic offenses (§ 24a German Road Traffic Act, StVG), while 32.8% were criminal traffic offenses (§ 315c and § 316 German Criminal Code, StGB). Cannabis use was predominantly detected in administrative offenses, whereas alcohol consumption played the major role in criminal traffic offenses. Among cannabis users, the majority were non-chronic consumers (79.2%). The main cause of accidents was self-inflicted loss of balance (59.5%; *n* = 22). Accident consequences were primarily minor injuries (67.6%; *n* = 25), with injuries occurring mainly to the face (*n* = 5), head (*n* = 6), and upper extremities (*n* = 5).

**Discussion:**

These data show that alcohol and cannabis consumption dominate the rising traffic offenses in Leipzig and its surrounding area. The results contain temporal and regional differences which point to distinct target groups and underscore the necessity of providing education regarding the legal framework and the impact of substances on driving and fall competences. In light of these findings, together with data from other regions, a re-evaluation of the statutory THC limit should be considered, as the current threshold primarily results in reduced penalties for occasional users, who may be at higher risk due to a lack of tolerance development. The observed injury patterns suggest an introduction of a helmet requirement.

## Introduction

E-scooters offer a flexible, cost-effective, and environmentally friendly way to travel short distances in urban areas. In Germany, these battery-powered kick scooters with electric motors can be used by people aged 14 and older without a driver’s license. Maximum speeds of up to 20 km/h are possible. The use of these scooters is regulated by the German Electric Micro-Mobility Vehicles Regulation (eKFV), which came into force on June 15, 2019 [6]. However, in the city of Leipzig, e-scooters have only become a permanent feature since the introduction of 35 rental stations by the Leipzig Transport Company (LVB) in October 2021, as the city’s mobility concept aimed to prevent scooters from being left indiscriminately in public spaces and thereby preserving the urban landscape [18]. Until May 2024, more than 500 vehicles were available with the increase of rental stations to 73 LVB stations (LVB correspondence) plus around 30 additional stations operated by other scooter providers. According to the Federal Statistical Office Germany, in the year 2024, the police recorded a nationwide increase of 26.7% reaching 11,944 e-scooter accidents with injuries, including a total of 27 fatalities [25].

With their growing prevalence, new challenges have emerged: police and regulatory authorities are increasingly recording violations by riders under the influence of alcohol or drugs, which occurs approximately two to six times more frequently compared to other modes of transport [20, 23, 25].

In terms of german traffic law, e-scooters are positioned between bicycles and small motor vehicles and, with regard to riding under the influence of alcohol and drugs, are subject to the same laws as drivers of motor vehicles [1]. These legal regulations are presented in Table 1.

**Table 1:** Overview of legal regulations for e-scooter users participating in road traffic

	Administrative offense	Criminal offense
Psychophysical impairments	not necessarily	yes
Abnormal driving behaviour	no	yes
Legal limit alcohol	0.5–1.09‰	≥ 1.1‰
Legal limit drugs	• Analytical limits of detection* • 3.5 ng/mL THC (since 22.08.2024)	-
Legal regulations	§ 24a German Road Traffic Act (StVG)	§ 315c and § 316 German Criminal Code (StGB)

* *THC* 1 ng/mL, *AMP-D* 25 ng/mL, *morphine* 10 ng/mL, *cocaine* 10 ng/mL, *benzoylecgonine* 75 ng/mL

In 2024, the partial legalization of cannabis through the Cannabis Act (CanG) entered into force on 1st April 2024. It legalizes the possession and private cultivation of cannabis by adults under destinct conditions. The aim of this reform was to curb the illegal market, strengthen health and youth protection, and to further regulate and decriminalize the handling of cannabis. At the same time, the commercial sale of cannabis remains restricted and subject to strict legal regulations.

In 2024, Germany introduced for the first time a statutory THC limit into its StVG. A limit of 3.5 nanograms of THC per milliliter of blood serum was added in § 24a StVG. Exceeding this limit constitutes an administrative offense when driving a motor vehicle. The new threshold replaces the previously applied non-statutory detection limit of 1 ng/mL. The regulation aims to create legal certainty and enhance road safety by distinguishing between residual THC levels from past consumption and actual impairment of driving ability. The law entered into force on 22nd August 2024. In addition, stricter provisions continue to apply: a zero-tolerance rule remains for novice drivers and drivers under the age of 21, and particularly strict prohibitions apply in cases of combined consumption of cannabis and alcohol.

The aim of this present study is to investigate alcohol and drug consumption among e-scooter riders in Leipzig and its surrounding area in the context of administrative and criminal offenses during the period from October 2021 to June 2024. A special focus will be laid on the regulatory changes referring cannabis consumption in 2024. In addition, an overview of accidents, their causes, and patterns of injury will be provided.

## Materials and methods

During the period from October 2021 to June 2024, the police recorded 411 e-scooter rides under the influence of intoxicating substances (alcohol and drugs) in the city of Leipzig and its surrounding area (predominantly in Torgau, Wurzen, Delitzsch). Cases without registration by the police, such as individuals presenting only to the emergency departments, were not included in this study. The reasons for conducting the testings were predominantly routine checks with no suspicious driving behaviour. However, our study also includes cases where suspicious driving behaviour or accidents with damage of property or physical injury were involved and which were therefore specifically targeted for inspection. The analysis of blood samples were carried out at the Department of Forensic Toxicology at the Institute of Legal Medicine of Leipzig, Universität Leipzig, Germany. For the present retrospective evaluation, data were stratified according to sex, age, type of offense, location and time of offense, toxicological findings, and accident details, which were recorded by the police accident unit. Further differentiation was performed according to the substance groups of alcohol, cannabis, amphetamine derivatives (AMP-D, including amphetamine [AMP], methamphetamine [MA], and MDMA), cocaine, and opioids (morphine, methadone). Besides blood analysis, only breath analysis for the determination of its alcohol concentration was carried out by the police officers in 66 cases.

To differentiate between mono-use of methamphetamine or amphetamine, as well as mixed consumption of both, the concentration ratio of MA/AMP was evaluated as shown in Table 2 [14].

**Table 2:** Overview of differenciation criteria of mono-use of MA and AMP and mixed use of both drugs

Concentration ratio MA/AMP	Assessment
≥ 5:1	MA-consumption (AMP metabolite)
3:1–5:1	Possible co-consumption of MA and AMP
≤ 3:1	Co-consumption of MA and AMP

Cannabis users were classified according to consumption frequency based on the detected THC-COOH concentration into four groups (Table 3) [30].

**Table 3:** Classification of cannabis consumption frequency according to concentration of THC-COOH

concentration of THC-COOH (ng/mL)	Assessment consumption frequency
< 37,5	occasional use
≥ 37,5–75	repeated use
≥ 75–150	regular use
≥ 150	chronic use

With regard to the partial legalization of cannabis in Germany under the Cannabis Consumption Act (KCanG) from April 1, 2024, the detected THC concentrations were retrospectively re-evaluated using the newly established legal threshold of 3.5 ng/mL for administrative offenses (§ 24a (1a) StVG).

The classification of offenses was carried out according to:

- § 24a (1) StVG for rides with blood alcohol concentrations (BAC) of 0.5‰ or higher, or a breath alcohol concentration (BrAC) of 0.25 mg/L or higher.
- § 24a (2) StVG for rides under the influence of other intoxicating substances (analytical thresholds of 1 ng/mL THC; 25 ng/mL AMP-D; 10 ng/mL morphine; 10 ng/mL cocaine; 75 ng/mL benzoylecgonine).
- § 316 StGB for rides with a BAC of 1.1‰ or higher or with the presence of psychophysical abnormalities.
- § 315c StGB for rides involving acute endangerment due to intoxication or psychophysical abnormalities.

Statistical data analysis was conducted anonymously using Microsoft Excel (Microsoft Office Professional Plus 2016, Redmond, Washington, USA) and IBM SPSS Statistics (Version 29.0.2.0, Armonk, New York, USA). Data were tested for normal distribution, followed by grouping into the respective categories. Subsequently, significance testing was performed at a significance level of *p* < 0.05 using the binomial test, Fisher’s exact test, chi-square test, Mann–Whitney U test, or Spearman´s rho correlation.

A positive ethical approval had been obtained to conduct this study (175/25-ek).

## Results

**Fig. 1:**
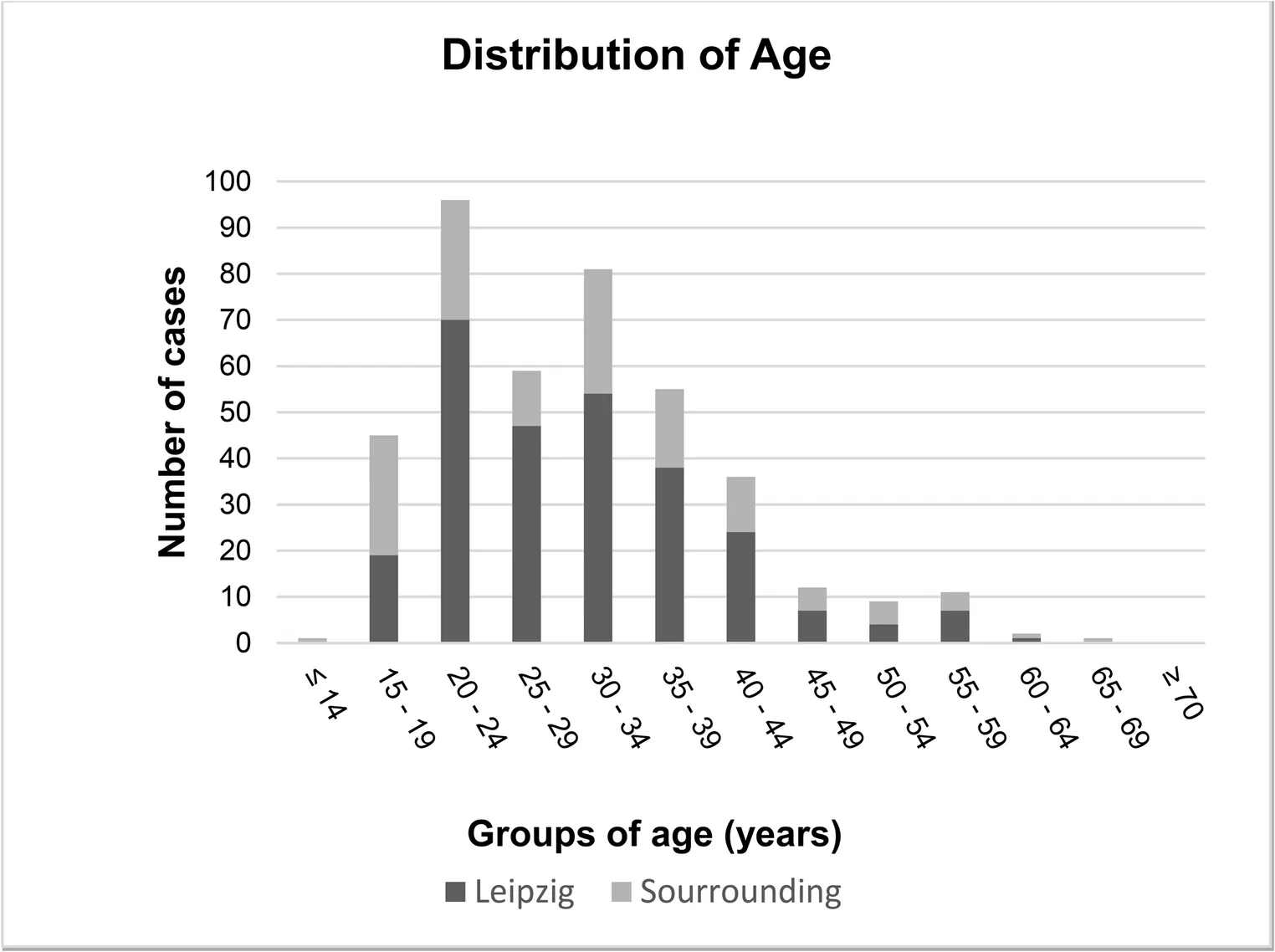
Age distribution of e-scooter drivers

The study cohort consisted of 91.2% (*n* = 375) male e-scooter riders. The mean age of the total study group was 30.6 years (range 14–67 years). The proportion of riders aged 15 to 19 was significantly higher in the surrounding area compared to the city of Leipzig (19.7% vs. 7.0%; *n* = 27 vs. *n* = 19; *p* < 0.01) (Fig. 1).

The majority of rides under the influence of alcohol and drugs (65.9%; *n* = 271) occurred in the city of Leipzig, while 33.3% (*n* = 137) were recorded in its rural areas (3 cases without information of location). In Leipzig, driving under the influence of alcohol was more frequently observed in the city center (45.1%; *n* = 73), whereas drug use among e-scooter riders was predominantly detected in the eastern part of the city (cannabis: 68.9%, *n* = 73; AMP-D: 65.6%, *n* = 42; cocaine: 72.2%, *n* = 13; and opioids: 77.8%, *n* = 7).

In the years 2022 (*n* = 83) and 2023 (*n* = 214), a significant annual increase of 157.8% (*p* < 0.01) in offenses was observed, with a higher incidence from March to October (73.7%; *n* = 219).

**Fig. 2:**
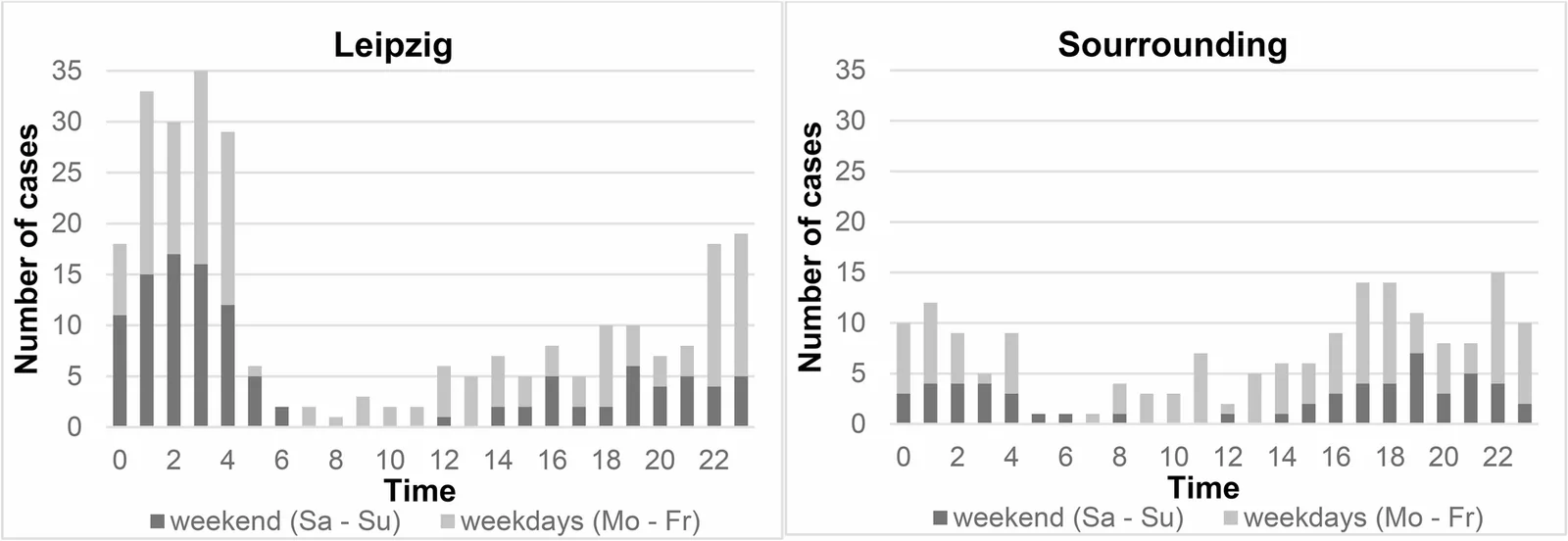
Presentation of the offense timing in the city of Leipzig and its surrounding area

In the urban area of Leipzig, rides under the influence of alcohol and drugs occurred primarily on weekends (42.8%; *n* = 116). In the surrounding area, this weekend trend was less pronounced (36.5%, *n* = 50). In Leipzig, weekday rides under the influence of intoxicating substances were concentrated between 10 PM and 5 AM (65.8%; *n* = 102), and on weekends between 12 AM and 5 AM (61.2%; *n* = 71). In the surrounding area, those rides were more evenly distributed, occurring mainly on weekdays between 2 PM and 1 AM (78.2%; *n* = 68) and on weekends between 4 PM and 4 AM (86.0%; *n* = 43). The weekday difference between the city and surrounding area was significant (*p* < 0.01) (Fig. 2).

Among the 411 rides under influence of alcohol and drugs, 421 legal violations were recorded, because 10 cases of mixed use of alcohol and another substance were assigned to both, § 24a (1) StVG and § 24a (2) StVG. During the study period, 283 administrative offenses were registered in 273 cases, of which 72.8% (*n* = 206) were assigned to the offense under § 24a (2) StVG. Criminal offenses accounted for 32.8% (*n* = 138) of violations, with alcohol involved in 98.6% (*n* = 136) of these cases. Of these, 90.4% (*n* = 123) presented alcohol-only consumption, while mixed alcohol use with THC, AMP-D and cocaine was identified in 12, three and one case(s), respectively. Only 1.6% (*n* = 2) of § 316 StGB cases involved mixed use of cannabis and methamphetamine (MA) without alcohol consumption (Table 4).

**Table 4:** Overview of legal violations

Legal violation	*N*		Alcohol mono-use		Alcohol mixed use		Other drug use	
	cases	%	cases	%	cases	%	cases	%
§ 24a (1) StVG	77	18.3	66	85.7	11	14.3	0	0.0
§ 24a (2) StVG	206	48.9	0	0.0	17	8.3	189	91.7
§ 315c StGB	16	3.8	13	81.3	3	18.8	0	0.0
§ 316 StGB	122	29.0	110	90.2	10	8.2	2	1.6
total	421	100						

In Leipzig, rides under the influence of alcohol occurred most frequently at 59.8% (*n* = 162), while in the surrounding area, cannabis predominated (48.2%; *n* = 66, Table 5). The proportion of positive alcohol detections was significantly higher in the city at 59.8% (*n* = 162) compared to the surrounding area at 40.9% (*n* = 56; *p* < 0.01). In contrast, the proportion of positive AMP-D detections in Leipzig was significantly lower at 23.6% (*n* = 64) than in the surrounding area at 32.8% (*n* = 45; *p* = 0.05). Opioid-related rides were recorded only in the city of Leipzig.

**Table 5:** Toxicological findings

		*N*	Alcohol	Cannabis	AMP-D	Cocaine	Opioids
Total	cases	411	220	173	110	28	9
	%	100	53.5	42.1	26.8	6.8	2.2
Leipzig	cases	271	162	106	64	18	9
	%	65.9	59.8	39.1	23.6	6.6	3.3
Surrounding	cases	137	56	66	45	10	0
	%	33.3	40.9	48.2	32.8	7.3	0.0
n/a	cases	3	2	1	1	0	0
	%	0.7	66.7	33.3	33.3	0.0	0.0

For rides under the influence of alcohol, single-substance consumption was observed significantly more often at 85.9% (*n* = 189). Cannabis was consumed alone or in combination with other substances in almost equal proportions. Mixed use was particularly noted in AMP-D (62.7%, *n* = 69) and in cocaine cases (96.4%, *n* = 27).

When considering the traffic-related administrative offenses (*n* = 273), the detection of alcohol consumption included 84 cases and was primarily determined by breath (65 cases, 77.4%) and secondarily by blood alcohol concentration (19 cases, 22.6%). Combined use of alcohol and other substances was present in 16 of these previously mentioned blood samples (84.2%). Administrative offenses were primarily associated with the use of cannabis (58.2%, *n* = 159) and AMP-D (38.5%, *n* = 105) (Table 6). Mixed use was present in 32.2% (*n* = 88) of the administrative offenses, noting that in cases involving breath-alcohol testing only, no assessment for the presence of additional substances was conducted, except for two cases showing a positive substance detection in the blood sample which were indicated by a positive DrugWipe test.

**Table 6:** Distribution of single and combined substance use

	**Substance**	**N**		**Mono-use**		**Mixed-use**	
		**cases**	**%**	**cases**	**%**	**cases**	**%**
Total (*n*=411)	Alcohol^1^	220	53.5	189	85.9	31	14.1
	Cannabis	173	42.1	75	43.4	98	56.6
	AMP-D	110	26.8	41	37.3	69	62.7
	Cocain	28	6.8	1	3.6	27	96.4
	Opioids	9	2.2	2	22.2	7	77.8
Administrative offences (*n*=273)	Alcohol^2^	84	30.8	66	78.6	18	21.4
	Cannabis	159	58.2	75	47.2	84	52.8
	AMP-D	105	38.5	41	39.0	64	61.0
	Cocain	27	9.9	1	3.7	26	96.3
	Opioids	9	3.3	2	22.2	7	77.8

^1^ Distribution: 154x BAC (29x mixed-use) and 66x BrAC (2x mixed-use)

^2^ Distribution: 19x BAC (16x mixed-use) and 65x BrAC (2x mixed-use)

**Fig. 3:**
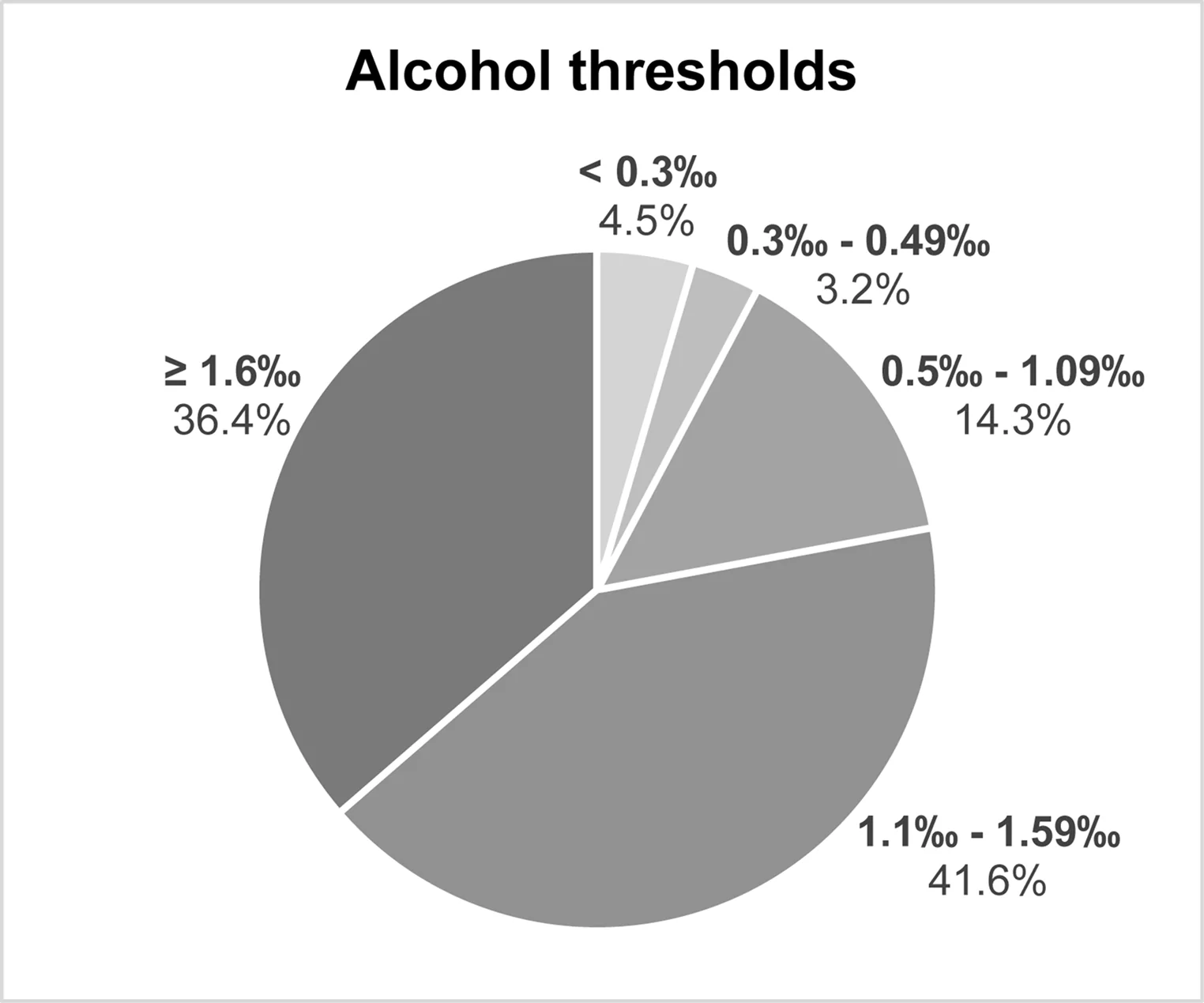
Distribution of blood alcohol concentrations according to alcohol threshold values

A mean blood alcohol concentration (BAC) of 1.42‰ was determined in the presented study cohort. In cases of alcohol-only consumption, the BAC was significantly higher, averaging 1.55‰, compared to 0.83‰ in cases of mixed alcohol use (*p* < 0.01) (Table 7). BAC above 1.1‰ and 1.6‰ were detected in 77.9% (*n* = 120) and 36.4% (*n* = 56) of the cases, respectively (Fig. 3).

In the present study population, alcohol users in the surrounding area were on average older (35.1 years) than those in the city of Leipzig (29.1 years; *p* < 0.01). Alcohol-related trips were recorded on weekends in 51.2% (*n* = 83) of cases in the city and 48.2% (*n* = 27) in the surrounding area. In Leipzig, these trips primarily occurred on weekdays between 10 PM and 5 AM (84.8%; *n* = 67) and on weekends between 12 AM and 6 AM (84.3%; *n* = 70). In the surrounding area, their timing differed significantly depending on the day of the week (*p* = 0.02): on weekdays between 3 PM and 2 AM (89.7%; *n* = 26) and on weekends between 10 PM and 4 AM (74.1%; *n* = 20).

Table 7 provides a summary of the substance concentrations detected in the blood of e-scooter drivers. Cannabis use was detected in the blood sample from 173 e-scooter riders (42.1%), of whom 75 individuals (43.4%) had not consumed any other drug. Stratification by consumption frequency showed a nearly even distribution across the four groups, with a declining trend from occasional to chronic consumption. The majority were non-chronic cannabis users (79.2%; *n* = 137; *p* < 0.01)). THC concentrations increased significantly with higher consumption frequency (*p* < 0.01). Mean THC concentrations were 3.14 ng/mL in occasional users, 7.32 ng/mL in repeated users, 18.12 ng/mL in regular users, and 31.34 ng/mL in chronic users (Table 7).

**Table 7:** Overview of detected blood concentrations of various substances

Substance	*N*	min.	max.	median	mean	SD
Alcohol	220					
only BrAC (mg/L)	66	0.24	0.61	0.40	0.39	0.09
BAC (‰) – total	154	0.0	2.80	1.37	1.42	0.57
BAC (‰) – mono-use	125	0.26	2.80	1.48	1.55	0.49
BAC (‰) – mixed use	29	0.0	2.35	0.77	0.83	0.54
Cannabis	173					
THC (ng/mL)		ca. 0.50	186.30	7.83	14.03	21.26
THC-COOH (ng/mL)		1.71	1190.90	63.00	100.87	129.71
*occasional consumption*^*1*^:	56					
THC (ng/mL)		ca. 0,50	16,40	2,00	3,14	3,10
THC-COOH (ng/mL)		1,71	34,90	15,75	17,04	10,16
*repeated consumption*^*2*^:	40					
THC (ng/mL)		2,53	15,70	6,67	7,32	3,31
THC-COOH (ng/mL)		37,80	73,00	53,55	53,97	10,02
*regular consumption*^*3*^:	41					
THC (ng/mL)		5,08	186,30	11,80	18,12	28,02
THC-COOH (ng/mL)		75,90	135,60	92,70	97,36	15,34
*chronic consumption*^*4*^:	36					
THC (ng/mL)		7.52	113.70	22.60	31.34	25.58
THC-COOH (ng/mL)		152.80	1190.90	235.70	287.36	180.52
Amphetaminederivatives	110					
Methamphetamine (ng/mL)	105	ca. 2.50	1400.80	264.80	343.35	307.23
Amphetamine (ng/mL)	16	11.80	322.60	78.45	101.30	83.27
MDMA (ng/mL)	9	10.70	385.00	39.60	100.46	131.32
Cocaine	28					
Benzoylecgonine (ng/mL)		ca. 2.50	1328.00	127.55	256.38	347.78
Opioids	9					
Morphine (ng/mL)	8	ca. 2.50	74.00	14.10	22.11	24.32
Methadone (ng/mL)	3	195.60	243.50	199.90	213.00	26.50

^1^ THC-COOH: < 37,5 ng/mL

^2^ THC-COOH: ≥ 37,5 < 75 ng/mL

³ THC-COOH: ≥ 75 < 150 ng/mL

^4^ THC-COOH: ≥ 150 ng/mL

**Fig. 4:**
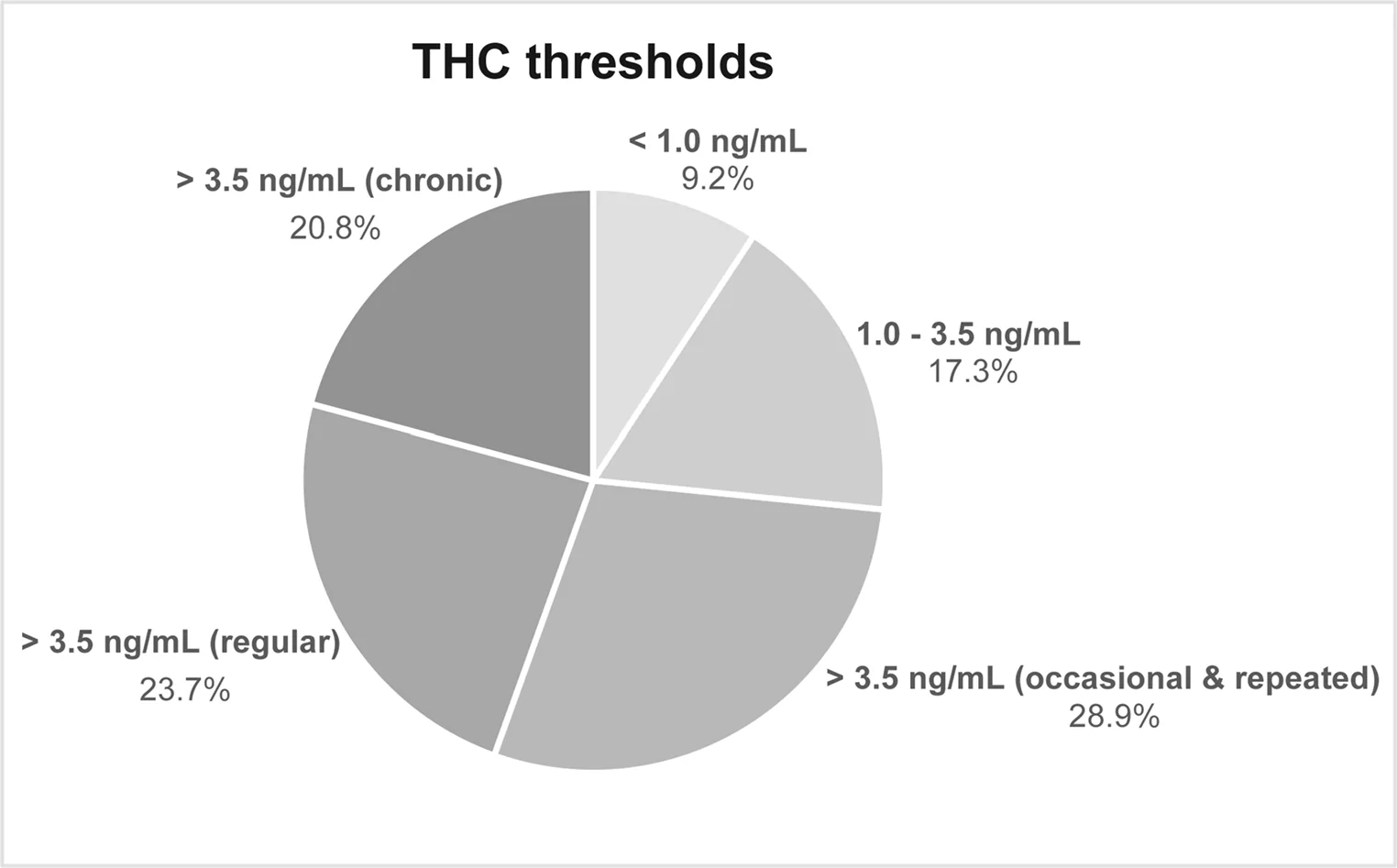
Distribution of blood THC concentrations according to THC threshold values

With respect to the partial legalization of cannabis in Germany (KCanG of April 1, 2024), no significant increase in cannabis-related rides beyond the general rise was observed. With the introduction of a legal cut-off of 3.5 ng/mL (§ 24a(1a) StVG, 22.08.2024) for THC concentrations in serum in cases of administrative offenses, there would have been 30 (17.3%) more cases below the legal threshold which would have not been punished in our study cohort. Further, 27 (90.0%) of these 30 cannabis users belonged to the group of occasional cannabis consumers (48.2%, *p* < 0.01), while 3 (10.0%) of them belonged to the group of repeated cannabis use (7.5%). However, in the groups of regular and chronic users even the lowest THC concentrations of 5.08 ng/mL and 7.52 ng/mL would have exceeded the new legal limit (Fig. 4).

Our data further show, that e-scooter drivers with especially high THC or THC-COOH concentrations took part in traffic. Table 8 shows five examples of exceptionally high cannabinoid blood findings, indicating both acute and regular cannabis use among the e-scooter riders.

**Table 8:** Examples of extraordinarily high cannabinoid concentrations in the blood of e-scooter users

THC (ng/mL)	11-OH-THC (ng/mL)	THC-COOH (ng/mL)
186.30	18.30	123.30
113.70	85.50	506.40
95.00	34.20	1190.90
73.60	24.00	236.70
71.20	24.80	273.10

AMP-D use was detected in 110 individuals (26.8%). Consumption of MA accounted for the largest proportion of 95.5% (*n* = 105). Of these, 11.4% (*n* = 12) involved mixed-use with AMP. In 23 cases (21.9%) mixed-use with AMP could not be ruled out. AMP-only consumption was observed in 3.6% (*n* = 4). Additionally, MDMA use was detected in the blood of 9 e-scooter riders (8.2%), of whom 88.9% (*n* = 8) had used it in combination with other amphetamine derivatives.

Both cannabis and AMP-D users in the Leipzig surrounding area were younger on average (26.9 and 30.7 years, respectively) than those in the city (30.3 years, *p* < 0.01; 36.6 years, *p* < 0.01). Furthermore, the mean age was 27.7 years for cocaine users and 38.9 years for opioid users. In contrast to alcohol-related impaired driving, the other substances were detected more evenly across weekdays and times of day.

In 9.0% (*n* = 37) of rides under the influence of intoxicating substances, accidents occurred predominantly in Leipzig (83.8%; *n* = 31) and less frequently in the surrounding area (16.2%; *n* = 6; *p* < 0.01). In all accidents, the rider was acutely under the influence of alcohol, with a mean BAC of 1.6‰. Most cases involved a BAC ≥ 1.1‰ (83.8%; *n* = 31; *p* < 0.01). Mixed-use with cannabis was present in 13.5% (*n* = 5) and with MA in 5.4% (*n* = 2) of cases (Table 9).

**Table 9:** Concentrations of mixed-use substances in the blood of e-scooter users during accidents

BAK (‰)	THC (ng/mL)	THC-COOH (ng/mL)	Methamphetamine (ng/mL)	Amphetamine (ng/mL)
2.35	1.69	32.40	24.20	4.60
1.52	11.10	109.30		
1.37	4.33	27.30		
0.49	6.10	128.30	43.80	8.96
0.12	5.27	65.60		

The main cause of the accidents was the self-inflicted loss of balance (59.5%; *n* = 22), followed by wheel locks (18.9%; *n* = 7), collisions with other vehicles (10.8%; *n* = 4), and collisions with stationary objects or pedestrians (each 5.4%; *n* = 2). Known accident scenarios (*n* = 30) occurred predominantly on dry weather (86.7%; *n* = 26; *p* < 0.01) and in darkness (63.3%; *n* = 19; *p* = 0.05). The consequences of the accidents were mainly minor injuries (67.6%; *n* = 25), less frequently severe injuries (18.9%; *n* = 7). In 5 accidents (13.5%), no personal injury occurred. Injuries primarily affected the face (*n* = 5), head (*n* = 6), and upper extremities (*n* = 5). The proportion of accidents among female drivers (27.8%; *n* = 10) was significantly higher than among male riders (7.2%; *n* = 27) (*p* < 0.01). Accidents occurred particularly on Saturdays (45.9%; *n* = 17), while otherwise following the general case distribution as mentioned earlier.

## Discussion

The presented study shows a comprehensive analysis of e-scooter-drivers under the influence of various substances in Leipzig and its surrounding area in Germany. 411 police-recorded e-scooter rides over a time period of 3.5 years were examined.

Our study cohort consisted of 91.2% male riders. In previously published German studies from Munich [7, 28], Hamburg [15], Würzburg [26], and Neubrandenburg [29], the proportion of male participants ranged from 83% to 98%. The average age of users in the present study was 30.6 years, which aligns with studies from Munich [16, 25], Hamburg [14, 15], Essen [20], Würzburg [26], and Berlin [27], with a range of 27 to 33 years. A small-town and rural study of 252 alcohol-related rides in the area of the Greifswald Institute of Legal Medicine reported an average age of 30.6 years [29], which is comparable to the present study in a metropolitan setting. However, the present study also showed that in rural areas, 13% more younger riders aged 15 to 19 used e-scooters. This may be due to regional differences in the availability of alternative mobility options for this age group. According to Gioldasis et al., individuals aged 17–24 exhibit increased risk-taking behavior regarding e-scooter rides under influence of intoxicating substances. In their study, over 40% of respondents reported having used an e-scooter at least once after consuming alcohol, and about 20% of them after taking other drugs [10].

The regional overall distribution of cases showed a clear majority of rides under the influence of alcohol and drugs in the city of Leipzig (65.9% vs. 33.3%). In 2024, the Federal Statistical Office reported that 53.7% of e-scooter accidents occurred in large cities with at least 100,000 inhabitants [25]. This can partly be explained by the availability of e-scooters, which are primarily rented in larger cities, whereas in rural areas they exist only as privately owned vehicles. Additionally, this observed distribution clearly reflects the capacities of police controls, which differ markedly between urban areas (primarily near “hotspots” with bars and restaurants) and rural regions. Similar findings were reported in Munich [7, 19] and Hamburg [15] where alcohol-related incidents were mostly observed near city centers.

The temporal development, showing a general increase in cases over the years and a higher incidence from March to October, on weekends, and during nighttime hours, corresponds to the results of studies from Munich [7, 19], Hamburg [15–17], Essen [20], Würzburg [26], Berlin [27], and Neubrandenburg [29]. The overall distributions in our study, with a concentration on weekends and during nighttime hours — particularly in the city — are most likely driven by the proportion of alcohol-related trips. As shown in previous studies [15, 17, 19, 20, 26], alcohol trips during this period are markedly dominant and, due to their large overall share, have a stronger impact in the city than in rural areas. Wudtke et al. reported that in rural areas, 78.1% of trips under the influence of alcohol occurred between 9 PM and 6 AM [29]. This temporal tendency is tempered in the overall analysis by other substance groups, which show a more even distribution across afternoon and evening hours. A similar temporal difference between alcohol-related trips and trips under other substances was also observed in Würzburg [26] and Melbourne [23].

As has already been shown in extensive analyses of traffic blood samples regardless of the type of vehicle [4, 30], there are regional differences in substance use due to various factors (availability, events such as Oktoberfest, police control density, and the diagnostic ability of police officers), which are also reflected in the blood findings of e-scooter users. In the present study, the following distribution of positive blood findings was observed: 53.5% alcohol, 42.1% cannabis, 26.8% AMP derivatives, 6.8% cocaine, and 2.2% opioids. After stratification into administrative offenses and criminal offenses, this distribution changes, as in nearly all cases of criminal offenses under § 315c StGB and § 316 StGB, alcohol consumption was detected. Among administrative offenses, cannabis use predominated (58.2%), followed by amphetamine derivatives (38.5%), alcohol (30.8%), cocaine (9.9%), and opioids (3.3%). Mixed-use was present in 32.2% of administrative offenses. Additionally, concealed mixed-use can be suspected, particularly in cases where only breath-alcohol tests were conducted. When restricted to administrative offenses, these cases accounted for 77.4% of the alcohol-positive cases, while the remaining administrative offenses confirmed alcohol via BAC, with 84.2% involving mixed-use. For rides under the influence of cannabis, no significant tendency toward mixed-use was observed, whereas amphetamines and cocaine were more frequently consumed in combination with other substances. In Würzburg [26], congruent findings were reported, with single-substance use observed in 44.6% of cannabis cases, 29.2% of alcohol cases, and 6.7% of amphetamine derivative cases. Between June 2019 and January 2024, there were 84 forensic blood analyses for driving safety assessments (excluding BAC > 1.1‰) which showed prevalences of 77.4% cannabis, 35.7% amphetamines, 28.6% alcohol, 10.7% opioids, 10.7% benzodiazepines, and 6.0% cocaine, with mixed-use present in 52.4% of the cases. In comparison, administrative offenses under § 24a StVG in Munich from early 2019 to mid-2021, based on 2,353 police recordings [7], showed 89.7% alcohol (6% BAC and 94% BrAC), 8.5% cannabis, 1.3% cocaine, 0.5% amphetamines, and 0.09% morphine, though likely for economic reasons, mixed-use with alcohol was not recorded, since alcohol alone was sufficient to constitute an offense. Meanwhile, 538 toxicological blood analyses [13] for administrative offenses during this period indicated single-substance prevalences of 47.6% cannabis, 31.6% alcohol, 1.7% amphetamines, 1.9% cocaine, and 0.2% heroin, with an undefined mixed-use in 17.1% of cases. The average blood alcohol concentration (BAC) detected in the present study was 1.42‰. In 78% of these offenses, a BAC above 1.1‰ was recorded. These data are comparable to findings from the regions of Munich [28], Hamburg [16, 17], and Neubrandenburg [29] (1.27‰ – 1.57‰, with 78–82% above 1.1‰).

Cannabis use was detected in 42.1% (*n* = 173) of the e-scooter offenses in the present study. The mean THC concentration in the present study was 14.03 ng/mL, which is comparable to the 65 cannabis cases from Würzburg [26] with an average of 14.6 ng/mL. Overall, the study clearly shows for the first time that non-chronic cannabis users (79.2%) predominantly use e-scooters. In their blood samples, an average THC concentration of 3.14 ng/ml (occasional users), 7.32 ng/ml (repeated users) and 18,12 ng/ml (regular users) was detected. Impairments in driving performance occur primarily after acute consumption. Occasional users may be similarly or even more severely impaired than habitual users, as they have less tolerance and less experience with the effects. Our study further shows clearly, that there are e-scooter drivers that consume cannabis either short before the ride or on a regular basis. A retrospective consideration of THC concentrations with respect to the in Germany newly introduced legal THC limit of 3.5 ng/mL (§ 24a (II) StVG) on August 22, 2024 for administrative offenses, shows that an additional 48.2% of occasional users would not have faced legal consequences. This demonstrates clearly, as previously shown for all road users in the regions of Leipzig [12], Cologne [22], and Rhineland-Palatinate [30], that even among e-scooter users, occasional cannabis users are less likely to be penalized under the new THC limit compared to more regular users.

Methamphetamine was detected in 105 blood samples. The median blood concentration was 264.8 ng/mL (mean 343.4 ng/mL), which is comparable to findings in the general traffic blood samples in the presented region (2022: 275.2 ng/mL; 2023: 299.1 ng/mL; 2024: 251.6 ng/mL; unpublished data). In the blood samples of e-scooter riders, particularly high MA concentrations of over 1000 ng/mL were also observed, indicating either acute use or tolerance due to regular consumption.

During the presented study period, 37 e-scooter accidents were recorded by the police. Data from Essen [20], however, indicate that a much higher proportion of e-scooter accidents (73.5%) are not reported to the police. Male users caused 73% of the substance-related accidents, consistent with analyses from Hamburg (70%) [15]. Previous accident data from various emergency departments in Hamburg [15, 17], Munich [19], and Berlin [27] showed an alcohol prevalence of approximately 20–40% without restriction to rides under the influence of intoxicating substances. In the present study, all accidents were associated with alcohol, with 84% of cases showing a BAC above 1.1‰. Mixed-use with cannabis or methamphetamine was detected in the blood of 7 e-scooter riders. Kähler et al. [16] similarly reported significantly more alcohol-related impairments at a BAC above 1.1‰. In an experiment by Zube et al. [31] involving 57 participants (51% male, aged 18–49, with experience using e-scooters and consuming alcohol), a 40% decrease in driving performance was observed at a BAC of 0.2–0.4‰, and a 72% decrease at 1.0–1.2‰. Wudtke et al. [29] observed a discrepancy between high alcohol consumption and few documented impairments, which was also noted in the present study and thus did not allow valid conclusions. Cannabis and methamphetamine were also associated with significantly more impairments [26], whereas in the present study, accidents occurred only in cases of mixed-use. Causes of the accidents were self-inflicted loss of balance, wheel locks (e.g. on curbs) or collisions with a distribution 60%, 19%, and 22%, respectively. In Berlin [27], a similar distribution was observed, with 57% of accidents due to loss of balance, while other studies from Hamburg [15, 17] reported 92% falls, showing a non-significant trend of more single-vehicle accidents under the influence of alcohol.

The injuries recorded in the present study were mostly minor (68%) and most frequently affected the head and face, followed by the upper extremities. Previous studies [15, 17, 20, 27] also found these two body regions to be the most commonly injured ones and observed that, compared with sober riders, alcohol-intoxicated riders sustained more severe injuries and more frequent involvement of the head region with a lower proportion of extremity injuries, suggesting reduced fall competence. In a study from Munich, the head and face accounted for 83% of at least moderate injuries (AIS ≥ 2) among e-scooter riders, which was higher than for bicycle riders. Additionally, 35% of e-scooter riders had a BAC > 0.5‰, significantly higher than the 16% observed in cyclists [11]. E-scooter falls are generally characterized by a lever effect due to the inertia of the low center of gravity, which can catapult the rider over the handlebars, creating a high risk of injury, particularly to the head and extremities [9]. The impact speed of the head even exceeded the standard limits required for bicycle helmets in Germany. DEKRA crash tests have shown that while helmets significantly reduce direct impact, rotational forces on the brain and neck largely remain [3]. In practice, Brisbane [21] observed a significant reduction in head injury risk following the introduction of a helmet mandate.

### Limits of the study

Due to the retrospective study design, there are limitations in data collection, resulting in partially incomplete documentation (e.g. helmet use, psychophysical impairments, precise injuries) that cannot be supplemented. Since the data were collected by the police, they are additionally subject to various filters and their plausibility can only be partially verified. It is impossible to ensure continuous monitoring and data collection at all times and locations, which is why police controls are concentrated at certain times and places. This can lead to biases in the results due to expectations and varying experience in handling and detecting substance use. For example, in cases where only breath alcohol tests were conducted, additional mixed-use of other substances may have remained undetected, as reliable verification would require blood analysis. Additionally, cases without police involvement, such as individuals presenting to emergency departments, were not detected. Furthermore, the new legal framework may cause a shift in prevalence and blood concentrations, particularly for cannabis use, which should be examined in future studies. In addition, populations naturally differ from one another, so the results are only partially generalizable.

### Summary and outlook

The present study shows that in Leipzig and its surrounding areas, primarily male e-scooter users are involved in traffic offenses, which occur predominantly during the summer months, on weekends, and in the evening and nighttime hours. Analysis of blood samples from these traffic participants primarily revealed alcohol and cannabis consumption. Non-chronic cannabis consumers mainly used e-scooters. Especially following the partial legalization of cannabis in Germany, there is a need to reevaluate the legal THC limit and raise public awareness regarding psychophysical impairments, which may be more pronounced in occasional users. Overall, further education on the legal regulations governing e-scooter use is necessary, particularly regarding intoxicated driving, which in Germany is regulated according to general motor vehicle legislation. Additionally, a helmet mandate should be reconsidered. In Germany, helmet usage rates have been recorded at 0–9% so far [2, 16], whereas countries such as Australia, Denmark, Greece, Italy, Croatia, and Barcelona already have universal mandatory helmet laws, and many other countries with association to age [8].

Further measures which are under discussion include adjusting the minimum age, introducing a driver’s license requirement, improving education and verification of traffic rules prior to e-scooter use, adapting speed limits, implementing nighttime riding bans, increasing wheel size, performing fitness-to-drive checks before rides (reaction tests, breathalyzer locks), as well as infrastructure expansion and routing over suitable roadways [2, 5, 19, 24, 27].

## Data Availability

The datasets generated and/or analyzed during the current study are not publicly available because we do not possess any freely accessible cloud storage but are available from the corresponding author on reasonable request.

## Abbreviations

AMP: Amphetamine
AMP-D: Amphetamine derivatives
BAC: Blood alcohol concentration
BrAC: Breath alcohol concentration
eKFV: German Electric Micro-Mobility Vehicles Regulation (Elektrokleinstfahrzeuge-Verordnung)
KCanG: German Consumption Cannabis Act (Konsumcannabisgesetz)
LVB: Leipzig Transport Company (Leipziger Verkehrsbetriebe)
MA: Methamphetamine
MDMA: 3,4-methylenedioxymethamphetamine
n/a: Not available
11-OH-THC: 11-Hydroxy-THC
SD: Standard deviation
StGB: German Criminal Code (Strafgesetzbuch)
StVG: German Road Traffic Act (Straßenverkehrsgesetz)
THC: delta-9-Tetrahydrocannabinol
THC-COOH: 11-nor-9-carboxy-THC
